# Coherent at face value: Integration of forest carbon targets in Finnish policy strategies

**DOI:** 10.1007/s13280-023-01923-3

**Published:** 2023-09-26

**Authors:** Samuli Pitzén, Jani Lukkarinen, Eeva Primmer

**Affiliations:** Finnish Environment Institute, Helsinki, Finland

**Keywords:** Environmental governance, Finland, Scientific knowledge, Policy coherence, Policy integration

## Abstract

Carbon sequestration and capture have gained a central position in forest governance, alongside wood production and biodiversity conservation, resulting in calls for policy coherence and integration across the EU. While coherence is often a target in the technical assessment of the policy design, it is important to understand how incoherent policies are supported by disconnected or incongruent knowledge claims and epistemologies. We address the coherence of forest policy by analysing the content and knowledge claims in forest, bioeconomy, and biodiversity strategies of Finland, an EU member state in which forests have a strong economic, political, and cultural status. Focussing on the argumentation regarding forest carbon, our analysis shows that the policy domains remain largely disconnected and rely on differentiated knowledge bases. Despite the explicit claims about policy coherence, few genuine attempts have been made towards integration and coordination between the domains. Our analysis reveals the different logics with which climate change is to be governed, and the types of knowledge utilised and produced in the integration of forest carbon as a policy object. Our analysis suggests that policy strategies with sectoral foci facilitate incoherent policymaking due to unresolved trade-offs and knowledge disagreements. Knowledge used in the policy design and implementation processes should be discussed thoroughly, and thereby integrated.

## Introduction

Coherence has been increasingly highlighted as a desired feature of policymaking, as it materialises in, e.g. the efforts of advancing cross-cutting policy objectives, such as the global sustainable development goals (UNPD 2022). According to the European Union (EU) terms, seeking coherence means that the EU and its member states need to reconcile economic, environmental, and social perspectives in environmental governance (Nilsson et al. 2012). To promote this objective, the European Green Deal calls for a new, more cross-sectoral perspective regarding environmental and climate governance. For example, the New EU Forest Strategy for 2030 explicitly advocates for coherent forest governance among member states, even though the forest policy domain is not strictly a formal competence of the EU (Aggestam and Giurca 2021; Lier et al. 2022). Indeed, the strategy seeks to improve synergies across the different functions of forests for delivering sustainability and climate neutrality (EU 2021). The growing importance of mitigating climate change as a grand societal challenge has made forest carbon a central issue of policy coherence, requiring integration. To achieve a coherent and attainable approach to governing carbon in forests, policies should also recognise the ecological boundaries of forests (Blattert et al. 2022). The nexus between biodiversity protection and climate mitigation has significantly grown, as highlighted by the shared actions of the scientific panels IPBES and IPCC (see Pörtner et al. 2021).

Nilsson et al. (2012, p. 396) define policy coherence as ‘an attribute of policy that systematically reduces conflicts and promotes synergies between and within different policy areas to achieve the outcomes associated with jointly agreed policy objectives’. Historically, the attempts to formulate common forest policies to promote more coherent environmental and climate policies in the EU have been met with resistance from member states with strong forest industries, such as Finland, France, Germany, Poland, and Sweden (Winkel and Sotirov 2016). At the same time, national forest policies are affected by various global and EU-level processes, such as environmental, energy, and water protection policies (Pülzl et al. 2013). For example, the United Nation’s Convention on Biological Diversity (CBD) and the EU Habitats Directive have been central policies driving biodiversity considerations into national forest policies (Harrinkari et al. 2017). Different EU strategies seek to offer cross-cutting approaches to governance and the promotion of policy coherence for environmental sustainability. To understand whether this is successful, attention needs to be given to national-level policy interpretation and objectives. Previous studies have identified differences in how member states comply with EU policies (Falkner and Treib 2008). Specifically, the role of knowledge production and science-policy interaction is becoming increasingly central in environmental policy processes that are also connected to forest governance (e.g. Saarela 2020; Ojanen et al. 2021; Rantala et al. 2022). A notable challenge in environmental governance is that scientific knowledge is used strategically to serve political purposes, rather than to relieve environmental disputes (e.g. Sarewitz 2004; Sivonen and Syväterä 2022).

There is dissimilarity in forest policies between the EU and member states with strong forest industries such as Finland and Sweden (Winkel and Sotirov 2016), which makes for an interesting context for our study. Compared to the other EU countries, Finland has a large volume of forest resources, a long history of their industrial use, and a legacy of institutionalised forest governance (Kotilainen and Rytteri 2011). Currently, Finland’s national forest policy is at a shifting point, with unprecedented pressure to consider climate and biodiversity objectives (Peura et al. 2022). Finnish forest policy has traditionally focussed on sustained yield and productive forestry, which has been challenged with demands to conserve biodiversity, resulting in responses seeking integration (Primmer 2011). Conservation demands have caused pushback in Finland, as they have been viewed as compromising timber production (Kotilainen and Rytteri 2011). The current call to simultaneously address climate and biodiversity challenges puts the sector policy into a new test on whether coherence between seemingly incongruent objectives can be attained in a country with forests that hold political, economic, and cultural significance. The previous Finnish government came to power in 2019 and committed to updating the sectoral strategies for forest, biodiversity, and bioeconomy policies spanning until 2030 and aligning them with the new EU policies. The timing offers a chance to analyse the strategies promoted during the previous policy cycle in their entirety. In order to meet the policy demand, the new strategies need to integrate new expectations to a policy landscape that is both strongly knowledge-driven and has high stakes for the Finnish forest sector. This is also reflected in the active public debate, frequent activist protests, and continuous media coverage around the attainment of climate targets. Thus, the Finnish policies offer an interesting case of how climate targets become politicised, also to be compared with other forest-rich EU member states.

Our analysis of *policy coherence in national forest, bioeconomy, and biodiversity strategies* examines how the different policy domains integrate forest carbon into their domain, the type of work executed in reconciling carbon-related objectives with other objectives, and what is left out of their argumentation. More specifically, we empirically analyse the three strategies and investigate *how they recognise the need to maintain forest carbon sinks and secure carbon sequestration, and how they adopt carbon as an object of governance.* In the next section, we present a theoretical framework of the link between knowledge, political interests, and governance.

## The role of knowledge production in policy coherence

Environmental governance has a close link to science and research-based knowledge. Different fields of science have laid the foundation for how environmental problems have been identified, framed, and understood during the last few decades (Wesselink et al. 2012). Governance mechanisms have been developed and adapted around new information relying on systems for ecological monitoring that make environmental processes identifiable for policy processes (Jokinen et al. 2018). Overall, environmental decision-making has been strongly linked to our evolving understanding of ecological processes across the globe. For example, findings regarding climate change have been actively used in political argumentation for global conventions to mitigate its effects (Saarela 2020). A recurring underlying assumption appears to be that producing more detailed information about ecological phenomena leads to better environmental governance.

The assumption about information improving governance has been contested by literature pointing to the discursive nature of politics, underscoring that policy discourses are not only informed by expertise and information, but they are also characterised by the interests of actors and formulated using various rhetoric measures (Stone 2002). Bocquillon (2018, p. 341) argues that collective action always involves the definition of problems, and that problems “do not wait out there to be discovered and solved, as rationalist and instrumental theories of policymaking posit. They have to go through a process of discursive construction to become problems in the first place”. Callon (1998) describes framing phenomena for governance as an act of creating and cutting connections between objects. Specifically, science and technology studies have drawn attention to how information production is not neutral, but *“*a process where the subject and object of information create each other in new ways*”* (Alastalo and Åkerman 2011, p. 28). The phenomena of creating governance objects and legitimising scientific knowledge for policymaking have been analysed, e.g. in the contexts of climate and biodiversity policies (Beck et al. 2014; Turnhout et al. 2014).

As pointed out by Leach et al. (2010), there are competing understandings of sustainability, based on diverging epistemologies and ontologies of different actor groups. In policymaking processes, actors carry out the epistemic work of promoting specific types of knowledge and excluding others to influence and shape people’s perceptions of reality, and to secure and grow support for their objectives (Alasuutari and Qadir 2014, 2019). A key point of debate in sustainability has been the possibility of decoupling environmental degradation from economic growth (see Gupta 2015). In this vein, Birch et al. (2010) note that bio-technoscience has been actively promoted as a solution to combining economic growth and environmental sustainability by increasing the efficiency of activities.

Utilising the Advocacy Coalition Framework (ACF) (Sabatier and Jenkins-Smith 1988, 1993), an analytical approach for analysing policy change and involved actors, Harrinkari et al. (2016) identify three coalitions attempting to shape the forest policy in Finland, namely a forestry coalition, an administrative coalition, and an environmental coalition. In their definition, the main priority of the forestry coalition is to maintain forestry and the forest industry as “lucrative businesses”, while the administrative coalition mainly focuses on the sustainable economic development of the forest sector, and the main goal of the environmental coalition is environmental protection (ibid.). During the recent years, the Finnish forest sector has participated strongly in the public discussions around the European Land-Use, Land-Use Change and Forestry (LULUCF) regulation implementing guidelines of the United Nations Framework Convention on Climate Change. Forest sector lobbyists have attempted to justify the increase in logging to a new high, even at the cost of reducing the national carbon storage (Sivonen and Syväterä 2022). This approach does not support Finland’s goals, nor the EU member states’ collective commitment, to take concrete actions towards carbon neutrality by utilising carbon sinks in the land-use sector to balance out the consumption-based carbon emissions. While the popular ACF approach focusses on specific stakeholder positions, strategies, and coalitions related to the policy preparation processes, it offers a less tangible orientation on the knowledge dynamics and operationalisation of the policy documents in a broader societal context.

Policy strategies are utilised to compile an overarching agenda for specific policy domains that attempt to steer policymaking, compile objectives, and map out how those could be reached. Strategy documents are examples of political rationalities, i.e. systematic ways to govern the society. Strategies define, e.g. policy targets, desirable technologies, and the rights and responsibilities of actors (Rose and Miller 2010). However, political rationalities do not constitute uniform ensembles, but rather the competing and conflicting approaches to governance (ibid.). Sectoral strategies have diverging objectives, and they are coordinated by authorities connected to different actor groups that have various views of how the forest policy should be practised (Winkel and Sotirov 2016). Therefore, the formulation of policy strategies may lead to conflicts and contestation, while their implementation may contribute to growing policy incoherence rather than well-aligned and harmonious developments (see Nilsson et al. 2012).

Policies may seemingly reconcile contradictory objectives until real-world limitations pit them against each other. Integration can be symbolic in that it is used as a legitimating rhetoric for policy processes and instruments that have no true integrative role (Winkel and Sotirov 2016). Moreover, various governance approaches have been strategically utilised to absorb the pressure to integrate biodiversity and climate policy in national forest policies (Sotirov and Storch 2018). Kröger and Raitio (2017) find that attempts to reconcile different interests between coalitions have created subsequent attempts to produce “more of everything”, leading to contestations in the policy implementation and the emphasis on a global “bioeconomy-productivism” discourse in Finland, where bioeconomy refers to a society that replaces fossil fuels with bio-based resources.

In bridging policy domains and solving some of the visible contradictions, the concept of policy coherence has attracted scholarly interest over the recent decades and spawned a growing body of research literature (e.g. Howlett and Rayner 2007; Nilsson et al. 2012; Carbone 2013). Most of these analyses are rooted in governance studies; they have, e.g. produced assessments of how coherent the policies are, along with analytical frameworks to assist in such undertakings (e.g. Nilsson et al. 2012; Makkonen et al. 2015). A central aim of these analyses has been to relieve conflicts between and within policies and policy domains. Another concept closely linked to policy coherence is policy integration. Policy integration refers to a process where a policy domain integrates the objectives originally addressed by other policy areas as part of their scope, attempting to align targets with one another (Jordan and Lenschow 2010). In the case of environmental policy integration (EPI), this refers to the consideration of environmental aspects in other policy areas, e.g. agricultural policy. The integration of biodiversity and climate policies has especially been recognised as a recurring policy challenge in the forest policy, as forests are tied to diverse livelihoods, policy domains, and economic sectors (Primmer 2011; Winkel and Sotirov 2016; Primmer et al. 2021).

Research on policy coherence has generally given more attention to policy implementation than policy formulation and agenda-setting (Righettini and Lizzi 2021). Moreover, while previous research has largely focussed on the pragmatic and technical aspects of policy coherence and integration—i.e. how coherent and integrated the specific policies are and how this can be measured—they have paid less attention to how policy coherence is connected to the utilisation and selection of scientific knowledge in policy formulation processes and their outputs. This is an important research gap, as Wesselink et al. (2012, p. 3) emphasise that ‘environmental discourses are not neutral descriptions of a real world out there, but are in practice based on human, and thus political or partial interpretations of technical knowledge by powerful interests’. In the context of Finnish forest policy, Sivonen and Syväterä (2022) have noted that representatives of specific policy domains give authority inconsistently to different scientific knowledge to justify their views regarding climate targets. In forest policy, policy integration generally has been shown to be hampered by sector interests (Winkel and Sotirov 2016), but the ways in which these different positions rely on specific knowledge domains have not been analysed.

## Forest policy context

European forest governance has traditionally made use of scientific knowledge (Farrell et al. 2000). Optimising forest growth and protecting forests from over-exploitation on the one hand, and degradation on the other have paved the way for generating inventory-based knowledge and mobilising expert advice. Finnish forest policy continues this legacy, featuring the shifting societal demands and interests during different stages of historical development (Kotilainen and Rytteri 2011). The institutionalised forest policy relies on scientific expertise codified in decision-making structures that have lately been challenged by more liberalised and market-driven developments (Kröger and Raitio 2017). Furthermore, the actions of forest owners are coordinated via expert advice (Primmer 2011), but their behaviour is more impacted by economic than ecological values (Valkeapää and Karppinen 2013; Deuffic et al. 2018).

The diverging science-based justifications for the roles of forests are reflected in the positioning of forests under different policy domains and their strategies at the EU level. For example, the EU’s Biodiversity Strategy for 2030 finalised as a part of the European Green Deal makes a strong stand on the biogenetic role of forests in climate mitigation policies by emphasising the strong interconnection between climate change and biodiversity loss (EC 2019, 2020a). Specific climate targets have been set to reduce carbon emissions by 55% compared to the level of 1990 by 2030, and reach carbon neutrality by 2050 (EC 2018, 2020b). These decisions amplify pressures on forests serving multiple roles in climate governance by maintaining carbon storages and sinks in the land-use sector, as well as contributing towards renewable energy targets in the Emissions Trading System and replacing fossil resources in the Effort Sharing Regulation (Lukkarinen 2017; EU 2018). The role of forests differs across the climate policy areas, which requires policy coordination and knowledge production to e.g. avoid the double accounting of emission reductions. Moreover, the climate mitigation role of forests becomes entangled with multiple sectoral policies, potentially leading to fragmented and contradictory recommendations (Sarewitz 2004).

The implementation of the EU-level policies and legislation is carried out by national governments and implementing agencies. The national contexts entail negotiating diverse—and oftentimes conflicting—interests, knowledge bases, and stakeholder views embedded in the industrial histories and socio-ecological conditions. In Finland, forests have been a major focus of three national strategies building projections regarding future demands and formulating plans for policy implementation—the forest strategy coordinated by the Ministry of Agriculture and Forestry, the biodiversity strategy coordinated by the Ministry of the Environment, and the bioeconomy strategy coordinated by the Ministry of Economic Affairs and Employment.

Our analytical approach builds on literature highlighting divergent understandings and epistemologies used in environmental governance in promoting coalitional interests and is tailored to serve our specific research interest. Based on the literature, we trace how the climate challenge is presented by different policy domains, how the challenge is to be governed, what knowledge claims are made, and what important aspects are left outside the problem-solving process.

## Methodology

Our empirical study followed a qualitative analysis of policy strategy documents informed by sociological interest in knowledge controversies (e.g. Foucault 2000; Gomart and Hajer 2003; Barry 2012). For the primary data, we used public policy documents outlining the official strategic objectives of three policy domains (forest policy, bioeconomy policy, and biodiversity policy) and their accompanying materials defining the more explicit targets, measures, responsible actors, and timelines (Table 1). These documents included the National Forest Strategy 2025 approved by the parliament in 2015 and updated in 2019, the Finnish Bioeconomy Strategy from 2014, and the Strategy for the Conservation and Sustainable Use of Biodiversity 2013–2020 from 2012 with an updated action plan from 2015. The selected strategies did not represent all the policy documents setting targets for the national forest policy, but they were the ones most actively referred to in the forest policy context, and they had European counterparts (Primmer et al. 2021). We excluded the Finnish Climate and Energy Strategy (Ministry of Economic Affairs and Employment 2017) from our analysis, as it directly focussed on climate policy and did not offer a chance to analyse how an independent policy domain integrates the climate policy as part of its objectives. The selected strategies included articulated targets and visions for forests and their use in the future, therefore allowing us to analyse the ways in which forest carbon is portrayed as a policy objective in different policy domains and assess policy coherence across these domains. The strategies also had different content areas, as the main focus of the forest strategy was on forest management and uses, the bioeconomy strategy on a market transition, and the biodiversity strategy on safeguarding and restoring natural ecosystems. Each strategy framed the mechanisms, responsibilities, and urgency of carbon sequestration differently.

**Table 1:** Primary and supplementary data used in the study

Primary data	Year	Referred to as	Other notes
Ministry of Agriculture and Forestry 2015. National Forest Strategy 2025. Ministry of Agriculture and Forestry 6b/2015.^a^	2015	The (Finnish/national) forest strategy	58 pages
Ministry of Agriculture and Forestry 2019. National Forest Strategy 2025—updated version. Publications of Ministry of Agriculture and Forestry 2019:17.^b^	2019	The (Finnish/national) forest strategy	128 pages
Ministry of Economic Affairs and Employment 2014. Sustainable Growth from Bioeconomy. The Finnish Bioeconomy Strategy.^c^	2014	The (Finnish/national) bioeconomy strategy	17 pages
Ministry of the Environment 2012. Luonnon puolesta—ihmisen hyväksi. Suomen luonnon monimuotoisuuden suojelun ja kestävän käytön toimintaohjelma 2013–2020 (In Finnish)^d^	2012	The (Finnish/national) biodiversity strategy	102 pages

Supplementary data	Year	Offered information about	Other notes
Policy workshop, Finland	2019	Roles of the strategies in forest policy	7 participants (Finnish)
		General policy developments	Policy actors
Stakeholder workshop, online	2021	Feedback to findings	36 participants (international)
		Coherence issues	Stakeholders
Dissemination event, online	2022	Feedback to findings	ca. 100 participants
		General policy developments	Forest specialists, scientists, government representatives
3 policy actor interviews	2019	Positions of different actors towards forest carbon policy	Total duration 169 min
		General policy developments	
		Coherence issues	
4 assessments related to the national strategies			
Antikainen, R. et al. 2016. Bioeconomy and cleantech in Finland—Assessment of Strategies and development suggestions. Prime Minister’s Office	2016	Assessment of the Finnish bioeconomy strategy	
Tanninen, T., I. Heikkinen, and M. von Weissenberg (eds.) 2017. Väliarvio Suomen luonnon monimuotoisuuden suojelun ja kestävän käytön strategiasta ja toimintaohjelmasta vuonna 2016. Ympäristöministeriön raportteja 14/2017	2017	Assessment of the Finnish biodiversity strategy	
Auvinen, A-P. et al. 2020. Impact Assessment of the Implementation of National Strategy and Action plan for the Conservation and Sustainable use of Biodiversity in Finland (2012–2020). Prime Minister’s Office	2020	Assessment of the Finnish biodiversity strategy	
Raivio, T. et al. 2022. National Forest Strategy 2025 Assessment (in Finnish). Gaia Consulting Oy, Pellervon taloustutkimus PTT	2022	Assessment of the Finnish forest strategy	
3 EU counterparts of the analysed strategies			
European Commission, Directorate-General for Research and Innovation 2018. A sustainable bioeconomy for Europe—Strengthening the connection between economy, society and the environment: updated bioeconomy strategy 2018. Publications Office	2018	EU bioeconomy strategy	
European Commission, Directorate-General for Environment 2021. EU biodiversity strategy for 2030—Bringing nature back into our lives. Publications Office of the European Union	2021	EU biodiversity strategy	
European Commission 2021. New Forest Strategy for 2030. COM/2021/572 final	2021	EU forest strategy	
https://www.biotalous.fi/aineistopankki/ website		Initiatives mobilised under the Finnish bioeconomy strategy	

^a^https://mmm.fi/documents/1410837/1504826/National+Forest+Strategy+2025/197e0aa4-2b6c-426c-b0d0-f8b0f277f332

^b^https://julkaisut.valtioneuvosto.fi/bitstream/handle/10024/161739/MMM_17_2019_National%20Forest%20Strategy%202025%20final_.pdf?sequence=1&isAllowed=y

^c^https://biotalous.fi/wp-content/uploads/2014/08/The_Finnish_Bioeconomy_Strategy_110620141.pdf

^d^https://ym.fi/documents/1410903/38439968/Luonnon-puolesta---ihmisen-hyvaksi.-Suomen-luonnon-monimuotoisuuden-suojelun-ja-kestavan-kayton-toimintaohjelma-2013%C3%A2%E2%82%AC%E2%80%9C2020-A1006DC3_DDD2_4710_AFD4_C0F29D96C110-31786.pdf/4b50b3a3-9301-9912-7dab-6b5481d4d573/Luonnon-puolesta---ihmisen-hyvaksi.-Suomen-luonnon-monimuotoisuuden-suojelun-ja-kestavan-kayton-toimintaohjelma-2013%C3%A2%E2%82%AC%E2%80%9C2020-A1006DC3_DDD2_4710_AFD4_C0F29D96C110-31786.pdf?t=1603260012095

We operationalised the concepts of policy coherence and integration by examining how carbon and climate policies were addressed in the strategies, and how consistent with one another their approaches to the governance challenge were. Policy strategies offer insight into the political argumentation, including problem definitions, knowledge claims, supporting evidence, set targets, and their implementation. As policy documents include the whole chain of argumentation, they also offer an opportunity to analyse the omissions. As argued by Valve et al. (2022), the comparative reading of policy documents can help to pinpoint ‘differences and interactions bypassed or abstracted from the separate accounts’. Policy strategies result from the policy formulation processes, maintaining the key parts that are necessary to understand their argumentation and evidence supporting their claims. In this, they reflect the consensuses and coalition-specific agenda better than the views of policy actors or parts of legislation. Moreover, strategies offer a chance to identify the degree of integration—i.e. what is being prioritised and what is included only rhetorically (see Pietarinen et al. 2023).

To gain more insights into specific policy continuums and implementation processes that the strategies are linked to, secondary data were utilised. Firstly, publicly available policy assessment documents and the official website for the bioeconomy strategy provided better insights on the concrete policy mechanisms mobilised under the strategies. Secondly, two specialist workshops and a dissemination seminar were organised in the years 2019–2022 to reflect views on the design and implementation of the policy strategies with responsible ministry representatives, implementing agencies, knowledge producers, non-governmental organisations, and companies operating in the forest sector. The workshops and interviews were primarily arranged for broader policy analysis (see Blattert et al. 2022, 2023). However, as the dataset included several important viewpoints explicitly on the climate policy role of forests, we used it to supplement the current analysis. The first workshop was recorded and transcribed, while notes were taken from the two latter events. Thirdly, policy actor interviews from 2019 to 2020 were used to further confirm and elaborate on the roles of the examined strategies and coherence issues. Interviewees were selected based on their expertise on forest ecosystem services to complement the views of the specialists attending the workshop. The interviews were transcribed verbatim. Finally, EU counterparts of the strategies were used in the analysis of significant exclusions (explained below).

We used a qualitative content analysis (Krippendorff 2004; Schreier 2012) to analyse the main references to climate change and carbon sequestration in the strategies, grouping them into systematic narratives and contrasting their differences. For the primary data, we started our analysis with a general reading to gain an idea of the overall logic and agenda of the documents. This task pointed our attention to the divergence of utilised scientific knowledge and differing epistemologies between the documents. Drawing from the literature described in the previous sections, our reading of the data, as well as our specific research interest of forest carbon, we created an analytical framework consisting of five analytical categories (see Table 2). Using the analytical framework, we analysed the content of the national strategies with a special focus on any references to climate and carbon using NVivo software. After this, we used content analysis to create descriptions of the content for each of the strategies in relation to climate policy integration. To guide our analysis, we used content analysis for the supplementary data to gain a wider understanding of the roles of the strategies, issues of coherence, and general policy developments. The supplementary strategies and assessments were also coded with NVivo software, while notes and transcriptions from workshops and interviews were analysed on Microsoft Word, as the analytical framework was not applied to them.

**Table 2:** Analytical framework

Analytical category	Main question(s)
Problem definition	How is the climate challenge defined? What (ecological and social) processes is it linked to?
Integration motivation	How is the policy objective made necessary?
Operationalised objectives	How will forest carbon be governed?
Knowledge claims	What are the important “facts” about forest carbon and its governance?
Significant exclusions	What problems or features of carbon are left outside the problem-solving process?

As the first category of our analytical framework, *problem definition* (see Bocquillon 2018) focusses on how climate change is understood by the policy domain, and to what societal processes it is connected to, guiding us to a better understanding of the underlying epistemologies informing the strategies. Secondly, we used the *integration motivation* category to analyse how national agenda-setting relates to other governance processes, industrial environments, and societal developments. This allowed us to understand the factors that called for the integration of carbon policies into the policy strategy (e.g. references to international commitments). Thirdly, the *operationalised objectives* category was used to capture the carbon-related policy objectives that the strategies presented and how they would be promoted, i.e. what role the policy domain would undertake regarding the climate policy. Along with these three analytical categories, we analysed the *knowledge claims* that the strategies presented as part of their argumentation for governing forest carbon, producing a general understanding of the role that knowledge plays in the justification for diverging governance approaches. Finally, we analysed *significant exclusions* (Asdal 2007; Barry 2020; Valve et al. 2022) to identify the questions and qualities of the object of governance and forest carbon that were excluded from the problem-solving process. The final category was operationalised by triangulating the three examined strategies to identify what key aspects present in some documents were missing or overlooked in others. This was supplemented by analysing the EU counterparts of the strategies to examine any key deviations between the national and EU strategies. For this, we selected the newest strategies publicly available while taking into consideration that, due to the policy development, newer strategies are likely to include more perspectives regarding the governance challenge. We used the analytical category to guide us to some, but not all, excluded aspects of forest carbon in the national strategies. The overall purpose of our analysis was to examine what logic informs the integration of carbon and climate policies as policy objectives, and what types of knowledge were utilised and produced in this process. Next, we move to presenting our analysis.

## Results

### Forest strategy

In the Finnish forest strategy, the general *diagnosis* of climate change was positive, with mostly desirable consequences and future developments that offered opportunities for developing the forest sector. The strategy described climate change as a long-term trend driving the transformation of the forest sector as a whole. Other major trends highlighting the need for renewing the forest sector included digitalisation, the importance of Asia as a trade partner, as well as increasing the focus on “responsibility” and “sustainability”. According to the strategy, our forest-related needs have evolved and become more diverse (via, e.g. the emphasis of recreational use, understanding the health benefits of forest environments, and the diversification of the forest industry’s economic structure), offering an opportunity to increase the overall wellbeing that forests may produce. Furthermore, climate change would further accelerate the growth of forests, which allegedly creates a positive impact on forestry. While the strategy recognised that climate change could also negatively impact forests, e.g. by bringing new plant diseases and pests, the emergent challenges were deemed solvable.

*The motivation for integrating climate and carbon-related objectives* into forest governance in the forest strategy relied on international legislation and commitments. According to the strategy, the LULUCF Regulation introduced the EU climate policy as an integral part of national forest policies, binding forests to wider climate targets. The strategy stated that Finland had committed to maintaining a carbon sink equivalent to 17–18 million tonnes of carbon dioxide, and to the Paris Agreement, which required carbon emissions to align with the amount of carbon that is being stored during the latter half of the twenty-first century. To stay within the 1.5 °C average of global warming, this objective should be reached by 2050. Another motive for the inclusion of forest carbon as part of the forest policy was the notion that the promotion of bioeconomy—referring to the utilisation of natural resources in a biological, sustainable way—would enable economic growth within the forest sector. In the forest strategy, the formal commitments on forest carbon balances set boundaries and justified active governance of forestry. However, the economic decoupling of emissions from production via bioeconomy was expected to relieve some of the ecological pressure on forests.

As *operationalised objectives*, the forest strategy proposed increasing the extraction of timber and other forest products while maintaining the minimum level of carbon stocks necessary to meet the climate targets of the Kyoto Protocol. The strategy relied on an estimated “highest sustainable harvest” that set the total level of national annual logging on the national scale. As a basis for the estimation, the strategy utilised calculations provided by the Finnish Natural Resources Institute. According to the strategy, the model included the consideration of technical–economic profitability and current restrictions from nature protection. However, the model did not include Finland’s carbon stock commitments for the years 2021 through 2030. While this model was presented as supporting evidence, the logging target in the strategy was not set at the maximum logging scenario of 85 million m^3^. Rather, the forest strategy sought to increase the national logging level to 80 million m^3^ in 2025, from 65 million m^3^ realised in 2013. During the same period, the strategy estimated a significant increase in the annual forest growth, from 105.5 million m^3^ in 2013 to 115 million m^3^ in 2025. As data for realised growth, the strategy referred to the National Forest Inventory (NFI) that only showed an increase to 107 million m^3^ by the year 2017. Therefore, the strategy assumed a significant acceleration in growth.^1^ The overarching objectives of the forest strategy were based on the assumed increase in the growth of forests, which would depend on the success of growth-promoting measures (explained below). It also assumed that climate change would positively impact growth while forest damages and decay could be controlled.

The forest strategy presented various knowledge claims regarding forest management practices as they are connected to carbon dynamics. Firstly, active rotation forest management, including the maintenance of a thinning cycle to allow select trees to reach their maximum growth, was promoted as a key strategy in maintaining the carbon sequestration capacity of forests. Secondly, new technologies and changes to societal practices, such as promoting entrepreneurship among forest owners, were expected to increase the efficiency of forest management in the forest strategy.

Finally, by governing sustainable levels of harvesting and governmentalising forest management, the forest strategy set the stage for climate mitigation through creating a market for wood-based products. On the one hand, this referred to extending the lifecycles of wood-based products, especially in construction materials that could act as long-term carbon storage, and substituting more carbon-intensive materials in products with shorter lifecycles. On the other hand, this referred to increasing the use of biofuels and replacing fossil-based energy sources. However, the latter was based on contested calculations that biofuels also would be considered a carbon–neutral energy source in the future. Overall, the forest strategy emphasised wood-based production chains in balancing the planned decrease in forest carbon stocks.

### Bioeconomy strategy

The guiding vision of the bioeconomy strategy was that transitioning to a bioeconomy would be a considerable economic opportunity for Finland. According to the strategy, rapid population growth, depleted natural resources, and diminished biodiversity called for a bioeconomy that was based on the production and use of renewable natural resources. Although the strategy imposed a global view on the “sustainability transition” of production systems (e.g. Bosman and Rotmans 2016), it carried a specific focus on the nationally important forest sector.

**Fig. 1:**
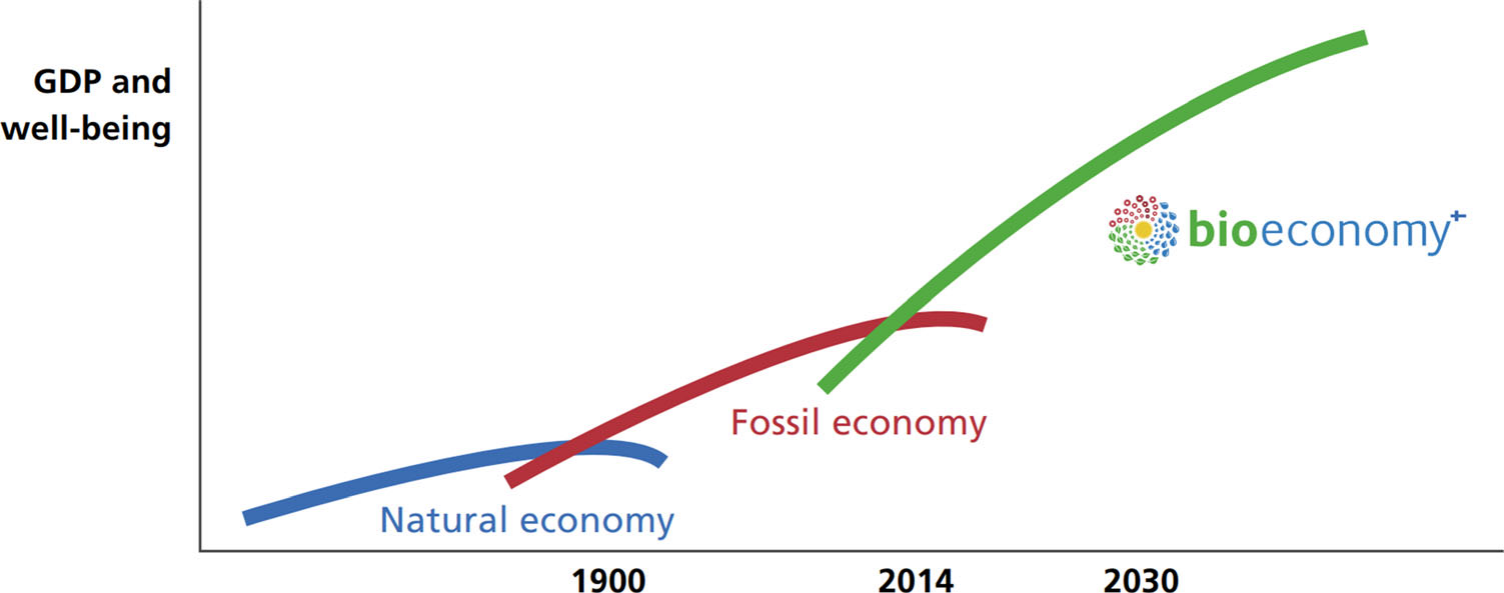
Bioeconomy presented as “the third major wave” of economic development. Retrieved from Ministry of Economics and Employment (2014, p. 5)

As the *diagnosis* of the climate challenge, the bioeconomy strategy portrayed climate change as an issue necessitating a transition to a low-carbon bioeconomic society, thus justifying its more specific objectives. The strategy introduced a future vision that featured more efficient usage of resources via new technological solutions and practices. The bioeconomy strategy emphasised that bioeconomic technologies and knowledge could offer Finland a chance for economic growth that would constitute “a third major wave of economic development”—the bioeconomy era, a leap from the preceding natural and fossil economies (Ministry of Economics and Employment 2014, p. 5). Due to the abundance of renewable resources, high-level skills, and industrial strengths, Finland was considered by the strategy to have a high chance of being a global frontrunner in the bioeconomy sector. In general, bioeconomy was portrayed as serving a national interest of growth in a rather linear and deterministic way. According to the strategy, bioeconomy could improve the national economy, employment, and wellbeing of citizens at an unparalleled level (Fig. 1). Regarding the supporting evidence for these claims, however, the strategy did not present studies or data pointing to such projected growth.

The strategy referred to bioeconomy as an important part of the EU’s general growth strategy. Moreover, its targets were deemed consistent with the UN’s Environment Programme and OECD’s definition of green growth. In addition, the EU bioeconomy strategy, as well as the related Horizon 2020 research and development programme, shared similar objectives seen in the Finnish bioeconomy strategy. The national bioeconomy strategy did not, however, refer to specific climate targets as *integration motivation* for the incorporation of forest carbon as an object of governance. Like the forest strategy, the bioeconomy strategy considered forests as a way of mitigating climate change by replacing fossil resources with wood-based construction materials and biofuels made from wood and forest industry side streams. The strategy did not include a more detailed discussion on the forests’ role in attaining climate targets.

The *operationalised objectives* that the bioeconomy strategy proposed were the promotion of bioeconomy, efficiency, and new technological solutions. Building on the same calculations used in the forest strategy, the bioeconomy strategy stated that the level of logging volume could be sustainably increased. Also, as wood-based biofuels were considered carbon–neutral, higher-intensity harvesting did not contradict the promotion of a “low-carbon society”. As the focus was on general industrial operations, the bioeconomy strategy had few concrete links to forest management. Moreover, the explicit *knowledge claims* on forest carbon remained on the general and systemic levels, where statements included little detail guiding specific actors.

### Biodiversity strategy

The vision of the biodiversity strategy was derived from the biodiversity objectives of the CBD and the EU Biodiversity Strategy aimed to halt biodiversity loss by the year 2020, and to ensure a favourable status of biodiversity and ecosystem services by the year 2050. The strategy stated that the protection of biodiversity was often overshadowed by other more immediate environmental problems, such as climate change. Therefore, to address its priorities, biodiversity loss was to be better connected to other environmental and societal dynamics, such as climate change. The strategy did not have economic growth as its focus, but rather emphasised controlling the environmental boundaries. Therefore, its *diagnosis* of the climate challenge was also the most reserved, with most precautions about future developments. Climate change was considered a threat to various biological processes, biotopes, and ecosystems due to gradual changes and more extreme weather events. Therefore, in addition to mitigating climate change, the strategy emphasised the importance of adapting to its effects.

The main *integration motivation* for climate change considerations was also derived from the CBD, in which climate change was presented as a global threat to biological diversity. Therefore, the maintenance of established carbon storages in forest ecosystems was expected to promote global carbon mitigation efforts. In other words, not only are healthy forest ecosystems operational carbon sinks supporting economic biomass circulation, but they also contribute to the ecological circulation—in which carbon storage plays an important role.

As the *operationalised objectives* of governing forest carbon, the biodiversity strategy promoted maintaining existing natural forest environments and restoring degraded ecosystems. As the strategy emphasised uncertainties related to climate change dynamics, the objectives were also connected to adaptation, the means for which were protecting vulnerable biotopes and enhancing natural recovery capacity and resilience. While the strategy presented no detailed calculations of the impacts of its objectives on carbon stocks and sequestration, it mobilised projects whose aim was to understand such dynamics better.

The biodiversity strategy also featured the *knowledge claim* that climate change would increase the growth of forests in northern regions. However, it presented reservations regarding the resilience and adaptation capacity of the domestic tree species in the face of rapid climate change and highlighted its reliance on genetic diversity. The strategy stated that imported foreign species had historically not succeeded well in Finland’s climate conditions. Therefore, the strategy maintained a precautionary orientation towards future developments.

Overall, the biodiversity strategy presented carbon dynamics as complex and only partially understood, following a precautionary principle. Thus, while concurring the view of increased forest growth, the strategy explicitly countered other views by stating that attracting economic activity to new areas also posed a threat to functioning biodiversity. Due to the more holistic ecosystem view, it did not describe explicit climate impacts of its proposed measures, but rather emphasised the systemic capacity to produce positive climate mitigation and adaptation outcomes.

### Cross-comparison of the strategies

The three policy strategies all employ distinct logics in the process of turning carbon into an object of governance. Based on our analysis of the strategies, as well as the supplementary data, the forest strategy relies on scientific estimations about the future developments and considers uncertainties solvable. Forest carbon is presented as an object of calculation that can be governed as an added element in the established management regime. In addition, mitigating climate change in forests is linked to processes that benefit the forest sector as a whole. The bioeconomy strategy, on the other hand, features techno-optimism in that new technological solutions and novel modes of operation will significantly relieve, or even solve complex socio-ecological challenges caused by climate change. The calculations and contestations regarding forest carbon dynamics are portrayed as a ‘black box’ to support the meta-narrative of a bioeconomy era as something qualitatively different from the earlier economic systems. Finally, the biodiversity strategy is the most precautionary and conservative out of the three, focussing on the risks of climate change and underlining the need to minimise disruptions to ecosystems and restoring their functioning. Furthermore, by portraying climate change from a risk perspective, the biodiversity strategy notes the necessity of active climate adaptation, and views the mechanisms of carbon sequestration as incompletely understood.

The three national strategies had some overlap in the knowledge claims that they put forward (Table 3). For example, climate change was considered to increase forest growth in the future in all of them. The forest and bioeconomy strategies framed this as allowing more economic growth via, e.g. increased logging. In contrast, the biodiversity strategy presented reservations regarding the adaptation capacity of domestic tree species and the effects of climate change on forest ecosystem services. Thus, it did not come to the same conclusion of climate change allowing more economic growth. From our interpretation, the main difference between the three strategies is the incorporation of a bioeconomy discourse in order to enable a growth orientation. While the forest and bioeconomy strategies shared a very similar understanding of growth potential, the biodiversity strategy did not even mention bioeconomy as a potential solution to the climate or biodiversity challenges.

**Table 3:** Cross-comparison of the strategies

	Forest strategy		Bioeconomy strategy		Biodiversity strategy	
	Rationale and measures	Knowledge claims	Rationale and measures	Knowledge claims	Rationale and measures	Knowledge claims
1. Problem definition	Growth-orientation; climate change will present solvable challenges and opportunities for growth in the forest sector	Climate change accelerates the growth of forests New risks (plant diseases and pests) can be managed The forestry sector, the management and use of forests, and the wellbeing derived from them will become more diversified, which offers great opportunities for the sector The sector can adapt to climate change, reducing vulnerability	Climate change necessitates a transition to a low-carbon society Growth-orientation; Technology and skills offer a chance of growth in the national bioeconomy sector	Bioeconomy is generally beneficial in a transition towards a low-carbon economy	Conservation orientation: climate change poses a serious threat to ecosystems	Climate change endangers various ecosystem processes, but its concrete impacts are yet largely unknown Climate change accelerates the growth of forests, which may cause negative consequences to biodiversity More research on carbon stocks and the carbon sequestration capacity of various ecosystems is needed
2. Integration motives	International commitments and regulation integrate climate policies to forest policies, especially combining timber extraction with carbon stocks Safeguarding the forest sector National competitiveness and economic growth	Due to planetary boundaries, consumption and production need to be sustainable Growth of the bioeconomy sector is growth of the forest industry If left unsolved, the effects of climate change could threaten forestry	National knowledge economy and economic growth	Technology and competence offer an opportunity for growth in the national bioeconomy sector	Safeguarding biological diversity, following the CBD	To safeguard biological diversity, climate change needs to be mitigated and adapted to
3. Operationalised objectives	Increasing the extraction of wood and wood-based products while committing to the minimum level of maintaining carbon stocks required by commitments Solving threats, such as plant diseases and pests, active monitoring and early reactions Diversification of the forest industry (incl. growth of the bioeconomy sector and circular economy)	Active forest management maintains the carbon sequestration capacity of forests Replacing fossil fuels with forest-based fuels mitigates climate change The efficiency of forest management can be increased, and the use of resources can become more efficient via bioeconomy and circular economy By increasing the lifecycle of wood-based products, carbon can be sequestered Biofuels will be considered non-polluting in the future	Promotion of bioeconomy and efficient and novel technological solutions	Activities can become significantly more efficient By utilising its biomass reserves, Finland can offer sustainable solutions to the mitigation of climate change and depletion of natural resources	Maintaining natural ecosystems and restoring degraded ecosystems Promotion of climate change adaptation Assessing and predicting the effects of climate change for nature	Protecting and restoring natural ecosystems is key to mitigating climate change and adapting to its impacts The most important measure of climate change adaptation is sufficient networks of nature conservation areas
4. Significant exclusions	Various uncertainties related to climate change, biodiversity Many negative future scenarios (threats deemed solvable) Preconditions for increasing the extraction of forest products on a large scale (forest owners’ willingness) The effects of increased extraction on biodiversity More efficient utilisation of wood products Alternative sources of income for foresters		Does not participate in the climate or carbon policy discourse on a detailed level Analysis of which actions are beneficial for carbon stocks and which are not Consideration of the negative environmental consequences of a bioeconomy		Does not explicate how Finland could attain its climate and carbon stock targets in forests No mentions about bioeconomy or increasing efficiency, or what consequences they would have for ecosystem functions No analysis of economic consequences of the proposed actions; instead, it aims at recognising the economic value of biodiversity and ecosystem services	

As a result of the cross-comparison between the three strategies, we found several *significant exclusions* they featured in the process of turning carbon into an object of governance. The forest strategy mostly overlooks various uncertainties regarding climate change and its implications for ecosystems. Only the positive future scenarios are presented in detail; therefore, the threats and uncertainties are considered solvable by targeted research and development projects. The forest strategy also mostly overlooks the biodiversity impacts of intensified logging, assuming the responsibility to be carried by nature conservation policies and legislation. It does not specify how intensified forestry could be compatible with the emphasis of the liberalised forest regime to ensure the freedom of choice of forest owners. The bioeconomy strategy excludes a detailed analysis and discourse regarding carbon dynamics in forests, deeming its objectives as generally beneficial. The biodiversity strategy does not explicate how Finland could attain its climate and carbon stock targets in forests but promotes research and development projects looking into carbon dynamics in more detail. Moreover, it does not mention what role bioeconomy and increased efficiency could play in attaining climate targets, and what potential or threats they might pose for biodiversity.

Comparing the Finnish bioeconomy strategy with its EU counterpart, we found that the EU strategy offers a more detailed view of how bioeconomy relates to ecosystems, noting that bioenergy production can also have negative environmental impacts. To this end, the EU strategy features a section that considers potentially harmful impacts of bioeconomy regarding biodiversity and the climate. Comparing the national biodiversity strategy with the EU equivalent, we noted a similar emphasis on synergies between biodiversity and climate policies. The EU biodiversity strategy argues that its objectives also benefit the climate, as nature and nature-based solutions are deemed essential for emission reduction and climate adaptation. Finally, the EU forest strategy had, from the forest carbon perspective, an emphasis on the efficient utilisation of wood-based products and promoting longer lifespans of forest products. Moreover, the EU strategy notes that incentive payments or the generation of tradable carbon certificates can offer foresters new sources of income; in the national strategy, it was mentioned that a potential price increase in emission allowance could increase the demand for biofuels. In doing so, the national strategy refers to the carbon market to argue for its objective to increase loggings.

The analysis showed that the national strategies had legitimacy to set an agenda within their sectoral boundaries with little inter-sectoral dialogue and coordination. The strategies do not present explicit policy incoherence, as they are directed to different societal audiences and dynamics. The forest strategy focusses on publicly and privately owned forests and their responsible management. Secondly, the bioeconomy strategy mobilises globalised sustainability discourses and technological solutions to forest-related industries and activities. And lastly, the biodiversity strategy is more concerned with the ecological developments taking place in situ within the ecosystems. Therefore, the different knowledge-based interpretations of climate change and forest carbon could, at face value, co-exist. In addition, the strategies promoted their agendas with minimal clashes by operationalising their targets via research and development projects. Therefore, the strategies could advance their specific ways to govern forest carbon in separate policy spaces.

## Discussion

Global conventions and evolving rules regarding the governance of mitigating climate change are forcing different policy sectors to react by designing carbon-motivated targets and actions, with an assumption that policies align towards a shared agenda (Di Gregorio et al. 2017). In the case of forest carbon, our analysis shows how climate mitigation targets are designed and operationalised in a forest-rich EU member state, Finland. Our analysis uncovers the ways in which national strategies most often referred to in the forest policy have adopted carbon as an object of governance. In the forest strategy, the forest carbon objectives are linked to the practice of defining sustainable levels of annual logging; in the bioeconomy strategy, to a techno-optimistic narrative of economic growth; and in the biodiversity strategy, to the complexities, risks, and adaptive capacities of functioning ecosystems. Drawing on the results of our analysis, we will present four discussion points: firstly, we discuss symbolic policymaking in the strategies; secondly, we assess the role of knowledge in policy incoherence; thirdly, we highlight the epistemic work carried out in the analysed strategies; fourth, to connect with societal discourse, we take a look at popular Finnish media coverage where the differing knowledge claims that the examined strategies have been built upon are debated. Finally, to draw the analytical message of the study, we discuss how the analysis of policy integration and coherence can benefit from a focus on knowledge practices.

Due to the increasing calls for coherence, policy strategies need to present themselves as comprehensive agendas, in which various aspects of governance challenges have been taken into consideration. This is expected to entail a dialogue with other policy domains, typically both as lengthy descriptions of the policy areas and widening the involved stakeholder groups (Larsen and Powell 2013). However, our analysis shows that the studied policy strategies work rather rigidly within their sectorial boundaries. Instead of effective measures for attaining climate targets and negotiating trade-offs within and between policy domains, the integration of climate policy largely appears as symbolic policymaking in the strategies (see Winkel and Sotirov 2016). While cross-cutting environmental phenomena would benefit from more integrated policies, our analysis points to siloed policymaking where the objectives and epistemologies of the strategies do not align. Such incoherence has significant material implications, demonstrated by the conflicts in implementing forest policy goals for climate mitigation and biodiversity protection that have recently gained significance in the European policy context (Blattert et al. 2023).

Scientific knowledge is central for the ways in which policy strategies integrate policy objectives to their policy domains. However, there are differing epistemic bases for the various knowledge claims and future projections that policy strategies present to legitimate governance choices. Knowledge claims are not politically neutral; in the previous analyses regarding Finnish forest policy, actors have been noted to give authority to scientific knowledge heterogeneously (see Sivonen and Syväterä 2022). Our analysis highlights that the analysed strategies present their pre-existing priorities as synergistic with the climate policy rather than adapt in the face of the governance challenge, pointing towards sectoral rigidity. Moreover, the strong narrative of decoupling (Gupta 2015) environmental degradation and economic growth employed in the forest and bioeconomy strategies is constructed on a techno-knowledge fix of the primacy of economic material streams in climate change mitigation, as recognised in the previous research (Birch et al. 2010). The contrast with the biodiversity strategy is stark, as it employs knowledge on the ecological complexity, risk, and balance of healthy ecosystems, and emphasises precaution. To overcome these sectoral barriers, more work should be done to connect the scientific bases, and reflect the epistemological choices and discrepancies, e.g. between economic evaluations and ecological assessments.

Our analysis shows that the epistemic work (see Alasuutari and Qadir 2014) carried out within policy domains is evident in the strategic inclusions and exclusions of policy objectives and their different aspects. This is shown in the significant exclusions the strategies make to avoid difficult questions, or disregard select perspectives of the governance challenge (see Asdal 2007; Barry 2020; Valve et al. 2022). Interestingly, the EU Emissions Trading System and carbon markets gain minor attention in any of the national strategies despite formulating a notable aspect of transnational climate policy framework. Moreover, the politics of knowledge in the strategies as shown by our analysis is based on black-boxing the issues of *what* is relevant regarding forest carbon when setting policy objectives, whether defining overall national forest growth, creating foundations for future bio-based industries, or protecting a functioning ecosystem. The analysis shows that integration suffers from little attention being given to the question of *how* the specified targets interact in practice and contribute towards the shared target of climate change mitigation. Therefore, improving the shared knowledge base across the policy domains is also important in improving the coherence of policy implementation and design.

Looking beyond the policy documents, the knowledge bases and assumptions regarding forest carbon have been questioned on several accounts in Finland, generating popular media interest and further highlighting the difficulty of creating coherent policy goals and societal consensus. We exemplify the debate with Finnish Broadcasting Company (YLE) news, which is openly accessible and covers the same stories on TV and radio. Firstly, in 2021, inventory results showing that the growth of forests had been less than expected compared to the projections were broadly covered in the news (Yle 19.10.2021). This puts into question the calculative assumptions about harvesting excess forest volume without compromising carbon stocks. Secondly, there is significant media coverage on the recent calculations by Statistics Finland, indicating that the LULUCF sector has, for the first time, been a net source of carbon emissions (Yle 25.5.2022). This has led to demands that the targets of the upcoming LULUCF climate plan need to be revised to accommodate more ambitious action. Thirdly, media reports have covered the statement by the European Parliament’s Committee on the Environment, Public Health and Food Safety (ENVI), saying that wood chips do not fill the requirements set for a renewable source of energy (Yle 28.5.2022). This further puts into question the presented role of biofuels as a key function for forests in climate mitigation. Our results endorse the idea that while producing and improving scientific knowledge is key in environmental governance, it alone is not sufficient in solving the wider sustainability challenge or incoherent and ineffective policymaking (Wesselink et al. 2012). Here, the public discussion in Finland mirrors developments in the neighbouring countries, such as Sweden, and shows how the politicisation of climate issues reaches to the operationalisation of scientific knowledge.

Our analysis examined policy strategies setting objectives for Finnish forests but has implications for policy integration and coherence across policy contexts and administrative levels. It is societally important to be reflexive on what roles policy strategies serve in mediating multiple interests, knowledge, and policy priorities. Strategy documents are outputs of policy formulation, collections of objectives within policy domains, taking on various roles that range from setting general policy objectives to realised plans for implementation. We argue that as policy strategies are calculated summaries of a sectoral agenda that contain comprehensive outlooks regarding political argumentation, they offer interesting data for the analysis of policy integration, coherence, and knowledge claims. Our analysis of the role that knowledge plays in policy integration and coherence shows that the examined policy domains use scientific knowledge strategically to serve their own interests as they present commitment to climate change mitigation. This notion is also highly relevant beyond the case of Finland, as similar challenges in integrating policy targets have been identified across the European countries with different characteristics (see Blattert et al. 2023). To facilitate more coherent governance, measures for negotiating differences between policy domains need to be more than symbolic. In forest policy, this requires the identification of trade-offs between different forest functions. To promote coherence, a truly cross-cutting approach to governance is needed to represent different values and help to re-integrate the fragmented knowledge domains. A prerequisite for such collective work would be the lowering of institutional boundaries for negotiation between the policy domains, supported with facilitated and coordinated approaches promoting mutual understanding rather than disconnected knowledge bases.

## Conclusions

Our analysis of how the policy strategies of three different policy domains in Finland integrate the management of forest carbon as a policy objective shows that the strategies set divergent objectives in the governance of forest carbon and avoid dialogue between governance approaches. The increasing pressure to mitigate climate change requires the policy domains to integrate and operationalise climate considerations in their agendas. This coincides with increasing calls for coherent environmental policymaking to improve the predictability and efficiency of actions across policy domains.

Previous analyses of policy coherence have largely focussed on policy implementation and analysing coherence in the realised policy instruments. In contrast, there is less of a focus on policy strategies representing outputs of policy formulation and agenda-setting processes. Our analysis finds that the strategies justify climate and forest carbon-related objectives with significantly different knowledge claims about climate change and future developments, building on pre-existing mandates of the policy domains. The attainment of climate targets is not prioritised in the examined strategies—rather, measures and objectives benefiting sectoral interests are framed as synergistic with the climate policy. The separate policy strategies operate as illustrations of the policy problem, but offer little in terms of bridging the policy domains so that forest carbon would be considered more holistically and coherently as a policy object.

Based on our analysis, we suggest three further research avenues on policy coherence. Firstly, the different epistemologies behind the politicised knowledge claims on forest carbon should be studied in more detail; how the knowledge bases of e.g. measuring (inventory), growth and yield, economics, ecology, and conservation sciences are reproducing the incoherencies of policy implementation, and what types of potential avenues for overcoming the epistemological rifts could be identified. Secondly, the strategic utilisation of scientific knowledge in policy processes requires further research, especially in the context of sustainability transformations connecting multiple socio-environmental challenges. While the case of forest carbon illustrates the complex knowledge dynamics that underpin the diverse stakeholder positions in environmental policymaking, such knowledge claims could very well exist in other policy areas, such as agriculture facing significant climate risks, the energy sector, or mobility systems. Finally, and most importantly, policy coherence requires the interconnection of environmental policy analyses and environmental science-driven trade-off analyses. This should be coupled with increasing reflexivity on the different ways in which scientific knowledge becomes mobilised in the policy processes. The other contributions in this special issue provide valuable science-based insights towards this end.

## Footnotes

1 With a stable 1.5 million m^3^ increase over four years, forest growth would increase by 4.5 million m^3^ by 2025.
